# Single and Mixed Feedstocks Biorefining: Comparison of Primary Metabolites Recovery and Lignin Recombination During an Alkaline Process

**DOI:** 10.3389/fchem.2020.00479

**Published:** 2020-06-05

**Authors:** Thomas Berchem, Quentin Schmetz, Thibaut Lepage, Aurore Richel

**Affiliations:** Laboratory of Biomass & Green Technologies, Gembloux AgroBio-Tech, University of Liège, Gembloux, Belgium

**Keywords:** pretreatment, biorefinery, biomass, multi-feedstock, *Cannabis sativa*, hemp, *Euphorbia lathyris*, lignin aggregation

## Abstract

*Cannabis sp*. and *Euphorbia sp*. are potential candidates as indoor culture for the extraction of their high value-added metabolites for pharmaceutical applications. Both residual lignocellulosic materials recovered after extraction are studied in the present article as single or mixed feedstocks for a closed-loop bioprocesses cascade. An alkaline process (NaOH 3%, 30 min 160°C) is performed to separate the studied biomasses into their main components: lignin and cellulose. Results highlight the advantages of the multi-feedstocks approach over the single biomass in term of lignin yield and purity. Since the structural characteristics of lignin affect the potential applications, a particular attention is drawn on the comprehension of lignin structure alteration and the possible interaction between them during single or mixed feedstocks treatment. FTIR and 2D-NMR spectra revealed similar profiles in term of chemical functions and structure rather than novel chemical bonds formation inexistent in the original biomasses. In addition, thermal properties and molecular mass distribution are conserved whether hemp or euphorbia are single treated or in combination. A second treatment was applied to investigate the effect of prolonged treatment on extracted lignins and the possible interactions. Aggregation, resulting in higher molecular mass, is observed whatever the feedstocks combination. However, mixing biomass does not affect chemical structures of the end product. Therefore, our paper suggests the possibility of gathering lignocellulosic residues during alkali process for lignin extraction and valorization, allowing to forecast lignin structure and make assumptions regarding potential valorization pathway.

## Introduction

The present study is included in the frame of the project “Tropical Plant Factory” that aims to rehabilitate disused industrial sites in Wallonia (south of Belgium) through the installation of indoor cultures. The targeted plants for the cultivation contain substantial amount of secondary metabolites that present a high potential for the pharmaceutical industry. ***Cannabis sp*. **and ***Euphorbia sp***. were selected in order to extract, respectively, cannabidiol (CBD) and ingenol derived compounds. The extraction of these compounds of interest generates significant amount of residual lignocellulosic materials as byproduct that could be used as raw material for further processes. Beyond the pharmaceutical interest of their secondary metabolites, *Cannabis sp*. and *Euphorbia sp*. have been recently depicted as potential feedstock for biorefining processes focusing on primary metabolites. For instance, **hemp shive** arouses interest in the frame of biorefining initiative because of its high polysaccharide content. This product is currently discarded as waste from the hemp industry. However, recent attempts have been made to valorize it as a potential source of monosaccharides to produce platform molecules through fermentation or via chemical reformations (Heo et al., 2019). To achieve this, those valorizations should be preceded by a pretreatment step that enables to reduce the granulometry and enhance the accessibility to cellulose fibers. Several kinds of pretreatments have been already performed on hemp shive. For instance, Gandolfi et al. (2015) performed acid organosolv on hemp shives prior to enzymatic saccharification and fermentation to produce lactic acid. Kuglarz et al. (2016) investigated dilute-acid as well as alkaline oxidative pretreatments to enhance the simultaneous production of succinic acid and ethanol. Moreover, Brazdausks et al. (2015) produced furfural and binder-less panels from hemps hurds by adding Al_2_(SO_4_)_3_ as catalyst to conventional steam explosion treatment. Finally, Semhaoui et al. (2018) combined the use of sulfuric acid as catalyst with a thermochemical process in order to improve the hemicellulose solubilization and the enzymatic hydrolysis of cellulose.

***Euphorbia lathyris***has been studied firstly as hydrocarbon-producing crops (or petrocrops) by M. Calvin in 1979 to substitute for crude oil, especially by extracting the terpenes and sterols from the latex. The cracking of *E. lathyris* allows recovering oil and cellulose convertible into fermentable sugars for ethanol production (Behera et al., 1995; Kalita, 2008; Jin et al., 2016). However, Abbasi and Abbasi concluded in 2010 (Abbasi and Abbasi, 2010) that Calvin's perspectives were not feasible, mostly due to the variations in yield and the necessity of a too wide land area to generate latex equivalent to a barrel of gasoline. Furthermore, *E. lathyris* has been investigated over the past decade for the production of non-edible oil. The plant is cultivated for the production of its rich-in-lipids seeds. The lipids are extracted and converted for biodiesel production. Some results demonstrated that *E. lathyris* is a promising feedstock and is a potential substitute since the lipid composition fits well with the EN 14124 standard (Wang et al., 2011; Zhang et al., 2018).

Both plant feedstocks are lignocellulosic material that need a crucial step to separate the polymers that rigidify the cell walls (i.e., cellulose, hemicellulose, lignin): the **pretreatment**. It is therefore worth briefly mentioning several methods for biomass pretreatment with advantages and drawbacks. **Organosolv** pretreatments rely on the treatment of the material with organic solvent or their aqueous solution mostly with the addition of acid or alkaline catalyst (Zhao et al., 2009). Organosolv treatment generate high purity lignin and allow to recover high quality cellulose and hemicellulose for further fermentation. The use of organic solvent, generally with low boiling point in order to ease the recovery step, implies the necessity of an efficient control of the process because of the fire and explosion hazard. Organosolv are currently considered as too expensive for biomass pretreatment at industrial scale. **Dilute acid** pretreatment is one of the widely use treatment in order to remove and hydrolyze hemicelluloses as well as enhance enzymatic accessibility of cellulose. Nevertheless, dilute acid treatment result in a poor delignification of the material (Schmetz et al., 2019). Moreover, mineral acids often used for these processes, are corrosive to the equipment and involve then the use of more robust material and higher cost of maintenance (Hayes, 2009). **Physicochemical pretreatments** including **steam explosion** and **liquid hot water** are another category of process that enables to remove hemicelluloses and increase the digestibility of cellulose. These processes present the advantage of having a low environmental impact, they do not involve the use of organic solvents and have relatively low investment cost (Chen et al., 2017). Nevertheless, physical treatments generate some inhibitory compounds for fermentation step and does result in poor delignification. Therefore, further treatments are needed to reach a more complete valorization of the biomass. Finally, **ionic liquids** are solvents for lignocellulose. They are considered as “green solvents” because of their low flammability, low vapor pressure, they are thermally stable and remain liquid on a wide temperature range. Ionic liquids allow to dissolve the whole lignocellulosic matrix and the selective recovery of cellulose, hemicellulose and lignin (Hayes, 2009). The recovered cellulose is less crystalline and more adapted to act as substrate for subsequent digestion. Even though these solvents stand amongst the most promising way of treatment for lignocellulosic feedstock, current ionic liquids present in some cases high hygroscopicity or moisture sensitivity as well as a high viscosity and are often corrosives because of their ionic character. Moreover, the synthesis of ionic liquids is currently too difficult and expensive to ensure their economic viability at industrial scale (Kunz and Häckl, 2016).

**Alkaline pretreatment** is known to enable high delignification of biomass, especially non-woody material, as well as an enhancement of cellulose enzymatic accessibility for further valorization (Kim et al., 2016). Briefly, alkaline pretreatment involves saponification of intermolecular ester bonds that result in a decrease in the crosslinking between xylan hemicelluloses and lignin of the raw material. The cleavage of acetyl and uronic bonds as well as glycosidic bonds in polysaccharides leads to a swelling of the material resulting in an increase of the specific area and the enzymatic accessibility of the polysaccharides (Chen et al., 2013). Along with these modifications, a reduction of the degree of polymerization and crystallinity of the cellulose are observed. Alkaline delignification depends mainly on the cleavage of aryl-ether bonds that are partly responsible of the crosslinking of monolignols. Alkaline treatment is known to affect especially α- and β-aryl ether bonds which constitute the major linkages in lignin (Xiao et al., 2001; Sanchez et al., 2011). Alkaline pulping using sodium hydroxide was assessed amongst four major lignin extraction processes (kraft, soda, organosolv and sulfite pulping). The study concludes that, economically as well as environmentally, kraft and sulfite pulping allow processing raw material at reasonable cost while organosolv pulping produce high quality lignin but involves high cost technologies. Finally, soda pulping is described as the more sustainable option as it generates low production cost and low environmental impact while being able to produce lignin for both low and high value applications as well as suitable carbohydrates for fermentation processes (Carvajal et al., 2016).

Nowadays, as the transition to a circular bioeconomy has become a central issue, it is essential to integrate “waste” valorization through a closed-loop bioprocesses cascade. The residue can be reused to produce biobased products and biofuels notably (Ronda et al., 2017). Currently, most of the lignocellulosic biomass conversion processes are studied or implemented on a single feedstock. Recently, an alternative approach was emerging that consists in mixing feedstocks from different resources. The combination is selected in order to reduce the costs by increasing the overall process efficiency, the yield and the productivity. In addition, availability of the material in a given location close to the facility or the collection point may influence the choice of feedstock (Oke et al., 2016). Thereby, this system could represent a solution in regions where insuring a constant and sustainable input of a single biomass might represent an issue. **Mixed feedstocks** have been recently considered in the ethanol refinery process (Althuri et al., 2017). Their influence on the processability of the products after pretreatment (diluted acid or steam explosion), the saccharification and the conversion of monomeric sugar into ethanol has been assessed. The resulting yields are similar to single feedstock (Shi et al., 2015; Oke et al., 2016). In addition, (Nielsen et al., 2019) reported some advantages to process multiple feedstocks in this field of applications. There is a beneficial economic impact due to less bulky infrastructures for storage and transportation. Mixed feedstocks allow also to increase the robustness of a process by limiting the variation within a single feedstock while remaining perfectly sustainable economically and technically (Michelin and Teixeira, 2016; Ashraf and Schmidt, 2018).

Beside the well-established use of polysaccharides, the economic viability of a biorefinery should ensure their economic viability through the valorization of lignin (Yamakawa et al., 2018; Dragone et al., 2020). In this economic context, there is interest to extract lignin from mixed feedstock to improve the profitability of biorefineries.

**Lignin** is, still today, considered as a “byproduct” and mainly burnt for its high calorific value (22.5–28.5 MJ/kg) where calorific value of cellulose is 14 MJ/kg (Demirbas, 2017). However, the unique structure and physico-chemical properties of lignin suggest potential high added values like antioxidant agents, surfactants, additives in the plastics processing or substitute in the phenol-formaldehyde resins. Moreover, lignin could be a raw material for the production of aromatic chemicals (phenols, benzene, toluene, and xylene) by means of depolymerization techniques (Zakzeski et al., 2010; Finch et al., 2012; Richel et al., 2012).

Lignins valorization perspectives are strongly dependent on their structural characteristics. As multi-feedstock treatments produce a single product from biomass A and biomass B instead of two products A′ and B′, it presents the risk of producing inhomogeneous lignin structure from one batch to another. That would not allow to maintain a sustainable production of constant characteristics product.

The present work highlights the feasibility of a multi feedstock refining of euphorbia and hemp compared to single feedstock process. The study focuses on the recovery step of the main components (cellulose, lignin, hemicelluloses) as well as the comprehension of lignin structure alteration and the possible interaction between lignins.

## Materials and Methods

### Samples

**Hemp** (**H**) (*Cannabis sativa* L.) was cultivated on fields in Marloie, Belgium by Belchanvre from 05/2016 to 08/2016, reaching a proper growth stage avoiding the accumulation of THC above 0.2%. The whole plant was let retting on field before Hemp shives and fibers were mechanically separated and stocked at room temperature in dry condition. **Euphorbia** (**E**) (*Euphorbia lathyris* L.) was cultivated by the Botanical Institute of the University of Liège in a greenhouse using earth as substrate from 08/2018 to 10/2018. The whole plant was dried at 40°C. Samples were pooled together to decrease heterogeneity due to the varying culture conditions. The two feedstocks were shredded into particles < 2.3 × 1.5 × 0.3 cm and dried at 50°C, then grinding to 0.5 mm particles was performed using a Fritsch PULVERISETTE 19 prior to alkaline treatment. A subsequent milling was carried out on the samples prior to composition analysis using a CYCLOTEC Tecator 1093 Sample Mill (sieve 0.5 mm).

### Treatments

Hemp shive alone (**H**), euphorbia alone (**E**) and a blend of 50/50 (w/w: Hemp/Euphorbia; **H/E**) were treated following an alkaline pretreatment using NaOH (Rossberg et al., 2015). First, 100 g of biomass samples were soaked in 1L 3% NaOH solution (ratio 1/10 w/v) and heated at 160°C for 30 min. The medium was let to cool down to 30°C then filtered through a 100 μm nylon filter. Liquid enriched in lignin was recovered and lignin was precipitated by decreasing the pH to 2 using H_2_SO_4_ (95%, VWR). The pellets were added to deionized water and dialyzed with a 1000 Da cutoff membrane for 4 days. Finally, freeze drying was performed using a FreeZone 4.5 (Labconco) for 3 days and stored in a dry place.

In order to investigate the behavior and the recombination of extracted lignin together, a second treatment step (same conditions: 3% NaOH (ratio 1/10 w/v); 160°C; 30 min) was applied on the extracted lignins (H, E, HE) to produce altered lignins H′, E′ and HE′. **Indulin AT (I**), a commercial G-lignin extracted from softwood by a Kraft pulping process was used as control to highlight new recombination with H and E lignin through new linkages not present in the native biomass. Mixed lignins produced from hemp or euphorbia and treated in presence of Indulin AT are noted, respectively, as follow (**HI**) and (**EI**). This experiment was performed in order to highlight eventual recombination of lignin with itself or with lignin from another feedstock. The second treatment is then assumed to act somehow as a purification step as it is supposed that a second treatment in fresh solvent is removing more impurities such as carbohydrates and proteins.

Each pretreatment process was performed at least in duplicate.

### Sample Characterization

***Moisture content***was quantified by mass loss after drying 1 g of sample in oven during 24 h at 105°C. A correction factor was applied to each sample to work on dry matter basis.

***Raw and treated biomass composition***was determined based on a NREL procedure (Sluiter et al., 2012). Briefly, raw samples were first exhausted by water and ethanol reflux during 3 days using a Soxhlet apparatus to remove extractible content. Then a hydrolysis was performed with 72% H_2_SO_4_ at 30°C for 60 min. A second step was carried out by diluting the medium to 4% with deionized water to perform a subsequent hydrolysis in autoclave at 121°C for 60 min.

***Klason lignin***was recovered by filtration through filtering crucibles, dried at 105°C and weighted. Combustion at 575°C for 4 h in a muffle furnace was performed in order to quantify residual ashes. Acid soluble lignins were neglected.

***Constitutive polysaccharides composition***was determined by measuring the monosaccharides content in the hydrolysate from Klason method after neutralization using CaCO_3_. Derivatization of monosaccharides into alditol acetates was performed as previously described (Schmetz et al., 2016) and analyzed by gas chromatography on a Hewlett-Packard (HP 6890) gas chromatograph equipped with a flame ionization detector. The monosaccharides were separated using a high-performance capillary column, HP1-methylsiloxane (30 m × 320 μm, 0.25 μm, Scientific Glass Engineering, S.G.E. Pty. Ltd., Melbourne, Australia). Glucose and xylose quantities were converted to the equivalent amount of polymeric glucan and xylan using anhydro corrections of, respectively, 0.9 and 0.88.

***Protein***content was estimated using a conversion factor of 6.25 based on the nitrogen content measured by the Kjeldahl method (Hames et al., 2008). Samples were mineralized and nitrogen was determined by titration using a Kjeltec 2300 (Foss).

Every compositional analysis was performed in triplicate.

### Lignin Characterization

Lignin purity was calculated using the Klason and Kjeldahl methods as described in 2.3.

#### Fourier Transformed Infra-Red

Chemical functions in extracted lignins were identified by obtaining FTIR spectra on a Vertex 70 Bruker apparatus equipped with an attenuated total reflectance (ATR) module. Spectra were recorded in the 4,000–400 cm^−1^ range with 32 scans at a resolution of 4.0 cm^−1^.

#### ^13^C-^1^H 2D HSQC NMR

Lignin units, linkages and contaminations were identified using Nuclear magnetic resonance (**NMR**) analyses were performed according to a protocol from Schmetz et al. (2019). 50 mg of lignin were dissolved in 750 μL of DMSO-d6. HSQC NMR spectra were recorded on a Bruker AVIII 400 MHz at 25°C. The spectral widths were 5,000 and 20,000 Hz for the ^1^H and ^13^C dimensions, respectively. DMSO peak was used as an internal chemical shift reference point (δ_*C*_/δ_*H*_ 39.5/2.49).

#### Thermogravimetric Analysis

Thermogravimetric analyses were performed using a TGA analyzer unit (Mettler Toledo) under a flowing nitrogen atmosphere. Approximately 10 mg of sample were heated in a porcelain crucible up to 800°C at a rate of 10°C/min (Manara et al., 2014).

#### Gel Permeation Chromatography (GPC)

In order to separate lignin fractions according to their molecular weight, a Agilent PLGel Mixed C (alpha 3,000 (4.6 × 250 mm, 5 μm) preceded by a guard column (TSKgel alpha guard column (4.6 mm × 50 mm, 5 μm) were connected to a HPLC system (Agilent 1200 series) equipped with a UV detector set at a wavelength of 270 nm. The lignin samples were dissolved at a concentration of 3 g/L in DMF with 0.5% of LiCl. The same solvent was used as mobile phase at a flow rate of 0.4 mL/min. 30 μL of each sample were injected on the system for a total analysis time of 40 min. Calibration curve was established with polystyrene standards from 1,000 to 30,000 Da (Sigma-Aldrich).

## Results and Discussion

### Sample Composition

*Euphorbia lathyris* (**E**) and hemp (**H**) shives (*Cannabis sativa*) dried powder were exhausted using a soxhlet apparatus leaving a lignocellulosic residue exempt of extractives. 36.3 % ±1.8 and 6.4% ±0.5 of extractives were removed with water and ethanol reflux from the E and H raw biomasses, respectively. It includes inorganics, proteins, soluble carbohydrates and the secondary metabolites such as CBD and ingenol mebutate. They are not taken into account as they are previously removed to be used for high value pharmaceutical applications as it is intended in a general approach of cascade valorization. The dry matter accounts for 95.2% ± 0.2 and 95.1% ± 0.1 for E and H, respectively. The major compounds constituting the biomass after extraction were determined on dry matter basis and are given in Table 1 in order to study the influence of the heterogeneous composition on the extracted primary metabolites (cellulose and lignin).

**Table 1:** Composition of exhausted biomass [hemp (H) and euphorbia (E)].

**Sample**	**E**	**H**
**Component**	**wt% dry basis**	
Cellulose	26.8 ± 2.5	28.1 ± 0.1
Hemicelluloses	15.8 ± 1.4	18.2 ± 0.6
Lignin (AIL)	15.1 ± 0.5	22.6 ± 0.9
Protein	10.8 ± 0.9	3.2 ± 0.4
Ash	5.0 ± 0.0	1.0 ± 0.2

Both samples are characterized by a similar content in cellulose and hemicelluloses meaning that processing a mix of these two biomasses would not change the total carbohydrate input in the case of a mixed-feedstock biorefinery. As hemicellulose is mostly composed of C5 monosaccharides (mostly xylose), the carbohydrate composition is quite similar.

However, Klason lignin (i.e., acid insoluble lignin) content is higher in H (22.6% ± 0.9) than in E (15.1% ± 0.5) which could decrease the accessibility of cellulose to treatment and impact the yield of lignin produced in a biorefinery dedicated, at least in part, to lignin valorization. This point is further developed in parts 2.3–6. In addition, proteins embedded in the structure (not extractable) account for three times more in Euphorbia (10.8%) than in Hemp (3.2%). As proteins tend to precipitate together with lignin after extraction, mixing heterogeneous feedstock will have an impact on both cellulose and lignin products.

### Mass Balance and Composition of Extracted Products

An alkaline pretreatment was performed on 100 g of a single biomass (H and E) or a 50/50 mix (HE) in 1L 3% NaOH solution at 160°C for 30 min. Results are analyzed in terms of yield of the different extracted compounds and regarding the quality and purity of the products.

**Figure 1:**
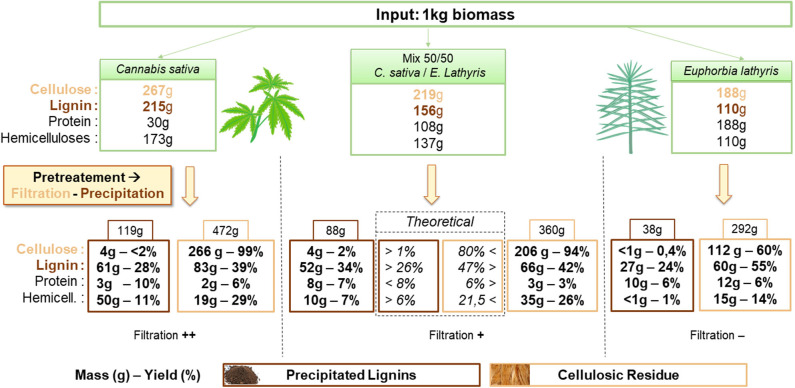
Mass balance of cellulose, lignin, protein and hemicellulose from *Cannabis sativa, Euphorbia lathyris*, and a 50/50 mix feedstock after NaOH pretreatment followed by filtration and precipitation.

The Figure 1 presents the mass balances resulting from the treatment on H, E and HE. Compounds yields are calculated on dry matter (DM) basis in the cellulosic residue (DM: E: 95.4%; H: 96.8%; HE: 96.7%) and the precipitated lignin (DM: E: 95.1%; H: 97.0%; HE: 93.7%). In addition, results from the mixed feedstocks treatment are compared to the theoretical average calculated from the results obtained from biomass treated alone. Firstly, as a filtration step is required to separate the cellulosic solid from the solution enriched in lignin (permeate), a first influence of the sample composition can be noticed. A significantly lower permeate flow is physically observed in the case of E compared to H. An influence of E composition is suspected notably from the higher content in protein (about 1/5 of the composition). Alkaline solutions at high temperature are known to extract efficiently proteins from biomass (Sari et al., 2015). The present process was able to extract more than 85% of total protein (>160 g) from E. Proteins in solution could promote pore plugging (Bolton et al., 2006). As a result, the filtration cake is assumed to retain more permeate that soaks the cellulosic residue retaining high molecular size compounds such as proteins and lignin. Lignin is then able to re-deposit on the surface of the fiber during cooling down and washing step (using water at pH 6.5). On the contrary, H shows good filtration properties in a way that mixing the two biomasses is likely to increase the permeate flow as H could act as a filtration adjuvant and decreases membrane fouling. Mixing the biomasses allows to recover more lignin from the permeate by precipitation as expected (34% > 26%) and 5% less lignin in the residue. This is probably due to the higher recovery of lignin from E that did not precipitated on the cellulosic residue during filtration. Recovery of cellulose in solid is also positively affected as an increase from 80 to 94% is observed compared to theoretical yield whereas the polysaccharides contamination in lignin is almost identical.

As the quality assessment of cellulose and lignin is essential to define the possible applications, the purity of pretreated biomass and precipitated lignin are presented in Table 2 and Table 3, respectively.

**Table 2:** Composition of pretreated biomass [hemp (H), euphorbia (E) and mixed (HE)].

**Sample**	**E**	**H**	**HE**
**Component**	**wt% dry basis**		
Cellulose	38.4 ± 3.4	58.8 ± 2.7	57.1 ± 0.3
Hemicelluloses	5.3 ± 0.4	10.7 ± 0.5	9.8 ± 0.2
Lignin (AIL)	20.6 ± 2.4	17.8 ± 0.0	18.3 ± 1.1
Protein	4.0 ± 0.7	0.4 ± 0.1	0.8 ± 0.1
Ash	22.5 ± 3.9	3.8 ± 1.6	7.1 ± 0.2
Total	90.8 ± 11.0	91.5 ± 4.9	93.1 ± 1.9

**Table 3:** Composition of recovered lignin [hemp (H), euphorbia (E), and mixed (HE)].

**Sample**	**E**	**H**	**HE**
**Component**	**wt% dry basis**		
Cellulose	1.9 ± 0.1	3.8 ± 0.3	5.6 ± 0.1
Hemicelluloses	1.8 ± 0.5	16.3 ± 0.8	11.5 ± 0.1
Lignin (AIL)	70.6 ± 0.9	51.6 ± 0.4	59.4 ± 0.5
Protein	27.9 ± 0.7	2.6 ± 0.3	8.9 ± 0.2
Ash	0.8 ± 0.1	2.0 ± 0.8	1.9 ± 1.6
Total	103.0 ± 2.7	76.3 ± 2.6	87.3 ± 2.5

At first sight, both H and HE biomasses are enriched in cellulose up to ~57% after treatment unlike E treated alone containing about 20% less cellulose. Lignin and ash are the main contaminants in the three samples. A non-negligible part of protein still remains in the pretreated biomass from euphorbia as discussed before due to poor filtration and high retention of liquid stream. The same observation can be drawn concerning lignin and ash being more retained on the E than H and HE. It can be noticed that hemicellulose content is decreased twice more in the case of euphorbia alone. Overall, mixed feedstock produces a similar cellulosic product than hemp alone while requiring a more efficient rinsing of the solid to avoid protein/ash accumulation due to the less effective filtration.

Concerning the lignin precipitated from the liquid stream (Table 3), E is characterized by a high percentage of protein (27.9%) and lignin (70.6%). However, proteins can artificially increase the amount of Klason lignin (Schmetz et al., 2019) which can misrepresent the real percentage and be responsible of the composition over 100%. However, it was neglected since applying a correction by subtracting protein content from Klason lignin would likely introduce an error greater than that caused by the presence of nitrogen compounds (Norman and Jenkins, 1934). The conversion factor to calculate the equivalent in protein from nitrogen content is unknown and difficult to assess in a degraded material such as Klason solid residue.

Mixed feedstocks treatment produces slightly higher contamination from cellulose but a greater quality lignin by decreasing hemicellulose and protein contamination compared to hemp and euphorbia, respectively.

### Insight on the Main Chemical Functions Present in Lignin Using FTIR

**Figure 2:**
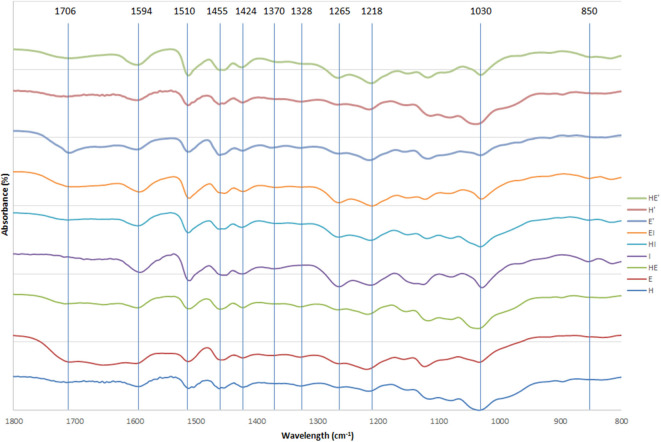
FTIR spectra of lignin and re-processed lignin (′) from Hemp (H), Euphorbia (E), Indulin (I), and mixed (HE, HI, EI).

Infrared spectra between 1,800 and 800 cm^−1^ of the different lignins are displayed on Figure 2.

Spectra of lignin samples were compared to assignments found in literature (Casas et al., 2012; Sammons et al., 2013; Gordobil et al., 2016; Domínguez-Robles et al., 2017; Qu et al., 2017).

The wide band around 3,300 cm^−1^ was attributed to aliphatic and phenolic OH groups and the signals at 2,935 and 2,870 cm^−1^ were due to symmetrical and asymmetrical CH, CH_2_. The bands at 1,708 cm^−1^ arising from non-conjugated carbonyl groups are more intense in samples after the second thermic treatment especially in euphorbia. The two peaks at 1,594 and 1,510 cm^−1^ were assigned to aromatic ring deformation while the signals at 1,455 and 1,424 cm^−1^ indicate the presence of C=C and C-H bonds in aromatic structures. The band at 1,370 cm^−1^, representative of phenolic OH and CH in methyl groups, is slightly accentuated after the second thermic treatment in every sample. Furthermore, the absorption band located at 1,328 cm^−1^ indicates the presence of C-O in S unit. A signal at 1,265 cm^−1^ attests to the presence of C-O in G unit, particularly in I lignin and mixed lignins containing I as well as in C lignin to a lesser extent. Different bonds in G unit (C-C, C-O and C=O) are represented by the signal at 1,218 cm^−1^. A weak signal around 1,120 cm^−1^, characteristic of a low amount of C-O-C bounds in alkali lignin, decreases between the first and the second thermic treatment, indicating a further cleavage of those linkages. Below this wavelength, the intense band at 1,030 cm^−1^ indicates the presence of primary alcohol not only in lignin, but also due to the presence of cellulose and hemicelluloses. Finally, the area below 1,000 cm^−1^ indicates C-H deformation. The band at 850 cm^−1^, representative of C-H in G units, is detectable only in softwood lignin (I) and mixed lignins containing I.

The same pattern can be observed in each lignin, indicating a similarity between the natures of the chemical structures of the samples. Nevertheless, qualitative differences can be observed between the relative intensity of the peaks. The relative intensities of the signals arising from mixed lignins (HE, HI and EI) are comprised between the relative intensities of the signals of the separate lignins they are composed of. This observation tends to indicate a mix of the two lignins rather than a recombination during the mixed feedstock pretreatment that would involve the creation of new bonds at the expense of others. This phenomenon would likely result in a significant change of proportion between the chemical functions.

### Elucidation of Structural Changes Through 2D-HSQC NMR

**Figure 3:**
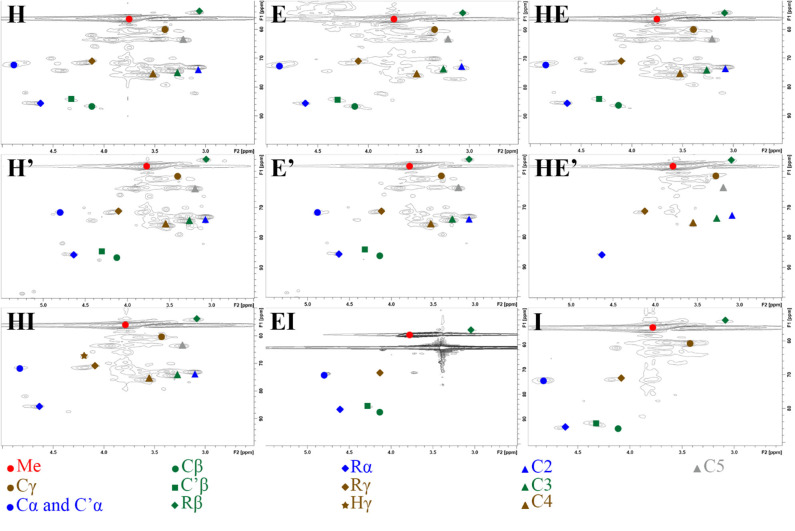
Side chain region (δ_C_/δ_H_ 50-90/2.5– 6.0) in 2D-HSQC NMR spectra of lignin and re-processed lignin (′) from Hemp (H), Euphorbia (E), Indulin (I), and mixed (HE, HI, EI).

**Figure 4:**
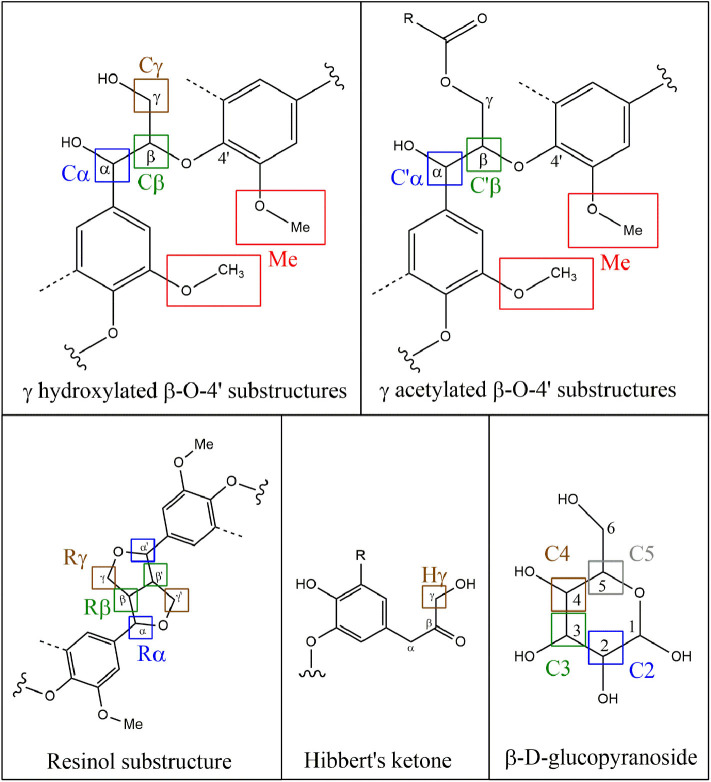
Representation of the main chemical structures in lignin observed in 2D HSQC NMR.

2D-HSQC NMR spectra provide, on one hand, similar results to FTIR showing relatively similar structures between lignin after the first and the second thermic treatment. However, in order to highlight a possible reorganization and a possible aggregation between lignin fragments, a close attention was drawn on the side chain region (δ_C_/δ_H_ 50–90/2.5– 6.0) (Figure 3) and the aromatic region (δ_C_/δ_H_ 100–135/5.5–8.5). To illustrate the 2D-HSQC spectra, corresponding structural elements of the lignin have been drawn in Figure 4. There was no structural information found in the aliphatic region (δ_C_/δ_H_ 50–90/3.0–5.0).

The most intense cross signal regardless the lignin sample was attributed to methoxyl group (Me; δ_C_/δ_H_ 55.80/3.75) (Yuan et al., 2011; Wu et al., 2015). Signals arising from Cγ-Hγ in γ hydroxylated β-O-4′ substructures (Cγ; δ_C_/δ_H_ 59.28-60.63/3.38-3.75) were identified as well as Cα-Hα in β-O-4′ and γ acetylated β-O-4′ substructures (Cα and C′α; δ_C_/δ_H_ 71.70/4.87), Cβ-Hβ in β-O-4′ and γ acetylated β-O-4′ substructures in S unit (Cβ and C′β; respectively δ_C_/δ_H_ 86.07/4.11 and 83.72/4.31) concerning ether bonds in lignin (Del Río et al., 2012; Kang et al., 2012). Concerning C-C linkages, Cβ-Hβ in resinol substructure (Rβ; δ_C_/δ_H_ 53.63/3.06), Cα-Hα in resinol substructure (Rα; δ_C_/δ_H_ 85.19/4.62), Cγ-Hγ in resinol substructure (Rγ; δ_C_/δ_H_ 71.01/4.17;71.05/3.77) (Liitiä et al., 2003).

The superposition of NMR spectra arising from lignins from single and mixed feedstocks, whether after the first or the second thermic treatment, did not reveal the formation of linkages of new kinds. The chemical bonds that are present in the lignin of both biomasses separately are present in lignin from mixed biomass as well. The same observation can be drawn between the first and the second thermic treatment. It can be underlined that few significant changes of intensity were observed between the NMR signals as well. However, it is worth noting that the second thermic treatment gave rise to Hibbert's ketone (Hγ; Cγ-H δ_C_/δ_H_ 67.57/4.20), attesting a more pronounced degradation of β-O-4′ linkages (Miles-Barrett et al., 2016). Even though Hibbert's ketone are usually formed during acid hydrolysis, a study released in 2013 showed that the production of this ketone could occur during the acidic precipitation of lignin (Narapakdeesakul et al., 2013).

HSQC NMR analyses highlighted the carbohydrates in lignin samples especially xylopyranoside in variable amount depending on the thermal treatment and the nature of the sample. Briefly, cross peak signals arising from C_5_-H_5_ in β-D-glucopyranoside (C5; δ_C_/δ_H_ 63.18/3.90 and 63.12/3.19), C_2_-H_2_ in β-D-glucopyranoside (C2; δ_C_/δ_H_ 72.66/3.06), C_3_-H_3_ in β-D-glucopyranoside (C3; δ_C_/δ_H_ 73.94/3.27) and C_4_-H_4_ in β-D-glucopyranoside (C4; δ_C_/δ_H_ 75.42/3.53) (Del Río et al., 2012).

The second treatment is performed with fresh alkaline solution that acts as a washing step, eliminating a significant amount of impurities that were remaining in the lignin fraction after the first thermic treatment. A quick glance at carbohydrates moieties NMR signals (δ_C_/δ_H_ 50-110/2.5-6.0) after the first and the second treatment highlights a decrease intensity of the signals arising from cellulose and xylose mainly in the blend E and to a lesser extent in HE while no significant change in intensity is observed in H lignin (Figure S1) (Kim and Ralph, 2014; Jiang et al., 2018; Wang et al., 2018). A similar observation can be drawn by comparing protein impurities signal in the region 4.1-4.8/50-60 of E lignin that are absent from E' spectrum (Figure S2) (Liitiä et al., 2003). A study on carbohydrates degradation under alkaline conditions confirmed a possible removal of remaining cellulose in the lignin sample since alkaline condition under 170°C promote the cleavage of cellulose in smaller fragments that are easily dissolved in the alkaline medium (Knill and Kennedy, 2002). The removal of the remaining proteins is also likely to occur as a prolonged alkaline treatment is considered as a common and efficient method to hydrolyze proteins into amino acids (Li et al., 2018).

### Thermogravimetric Analysis

**Figure 5:**
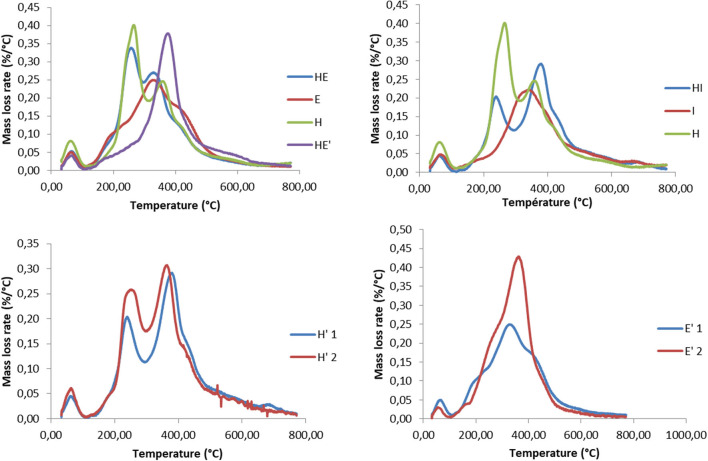
Thermogravimetric analyzes of lignin and re-processed lignin (') from Hemp (H), Euphorbia (E), Indulin (I), and mixed (HE, HI, EI).

Results of the thermogravimetric analyses are shown in Figure 5. The change in sample mass below 120°C was attributed to moisture and is therefore not displayed on graphics. Thermal degradation of every samples begins slowly at 120°C, then, DTG curves present a first, intense, degradation peak between 210°C and 300°C for the HE, H and I lignins and a small shoulder between 170°C and 230°C for the S lignin. This decomposition is assumed to be mainly linked to carbohydrate, and especially hemicelluloses and amorphous parts of cellulose (Manara et al., 2014; Wang et al., 2019).

More crystalline structures of cellulose are decomposing at higher temperature, on a range comprised roughly between 300°C and 400°C (Yang et al., 2007). Degradation of lignin occurs on a wide temperature range, between 250°C and 600°C. Low temperature degradation (up to 300°C) is reported as the cleavage of β-O-4 bonds while more stable bonds, namely C-C and β- β, are cleaved from 500 to 600°C (Moustaqim et al., 2018). A small degradation beyond this point is likely to be attributed to condensed lignin. The present DTG curves is in agreement with the composition of the lignins, since the hemp lignin is the one containing the highest rate of carbohydrates, mostly hemicelluloses and Indulin is the purest lignin among the samples and exhibit the lowest amount of carbohydrates degradation peaks.

Firstly, it can be noticed that the weight loss attributed to structural carbohydrates and proteins decrease after the second thermic treatment, regardless the nature of the sample. This could be explained by the process itself. Since the second treatment is performed with fresh alkaline solution, this process can act as a washing step as well, eliminating a significant amount of impurities that were remaining in the lignin fraction after the first thermic treatment.

An increase in the degradation temperatures can be observed between the first and the second thermic treatment. This change of behavior is attributed to a higher proportion of more resistant chemical linkages such as C-C bounds. It is supposed that, during the treatment, lignin is firstly fragmented into soluble oligomers, especially through the cleavage of C-O-C ether bonds while re-polymerization reactions occur with the ongoing of the reaction (Rößiger et al., 2018). It was shown on model compounds that depolymerization compete with re-polymerization reaction. The latter favor the formation of more resilient linkages than the initial C-O-C ether. The re-polymerization is believed to be all the more important that large lignin fragments are large since cross-linking of phenolic units increases the proximity and the orientation effects (Kozliak et al., 2016). The degradation of β-O-4′linkages during the second thermic treatment is underlined by the detection of Hibbert's ketone by the 2D-HSQC-NMR analysis (cf. part 2.1 2D HSQC NMR).

### Monitoring the Molecular Weight of Lignin Polymer by Gel Permeation Chromatography

Both lignins recovered after the first and the second treatment have been then analyzed by gel permeation chromatography to have a better insight on the variety of sizes that can be obtained through the present process. Three major outcomes can be drawn from these results. First, it can be noticed that E, H and HE lignins are all characterized by major polymers around 5,600 and 4,000 Da as seen in Table 4. The superposition of the GPC chromatograms of HE lignin from the first thermic treatment does not point out any aggregation between H and E lignins.

**Table 4:** Molecular weight (M_w_) of lignin and re-processed lignin (′) from Hemp (H), Euphorbia (E), Indulin (I), and mixed (HE, HI, EI).

**M${\text{M}}_{\text{w}}^{*}$**	**Max**	**P1**	**P2**	**P3**	**Min**
**Samples**					
H	130 000	/	5 700	4 000	400
H′	190 000	13 400	/	/	200
E	60 000	/	5 600	4 000	460
E′	170 000	11 000	1 700	300	30
HE	140 000	/	5 600	4 000	900
HE′	130 000	16 000	/	/	300
I	150 000	21 000	/	/	900
EI	150 000	15 000	6 000	/	600
HI	150 000	12 000	6 000	/	750

^*^M_W_ are calculated for each peak (P) and from the first (max) to the last (min) signal on the GP chromatogram.

**Figure 6:**
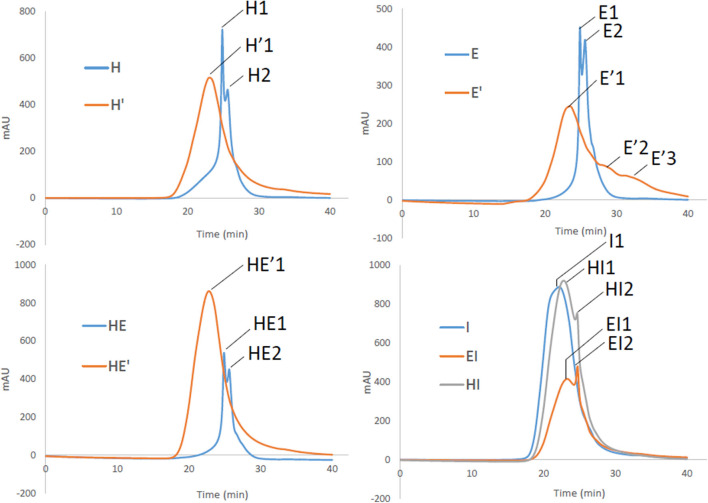
Gel Permeation chromatograms of lignin and re-processed lignin (′) from Hemp (H), Euphorbia (E), Indulin (I), and mixed (HE, HI, EI).

However, reprocessing the lignin led to a shift of the major polymers to a unique broader distribution around polymers greater 10 000 Da in the case of H′ and HE′ as depicted on Figure 6. Notably, E′ lignin shifted as well to higher molecular weights (11,000). Additionally, the second treatment promoted as well the cleavage to lower size polymers around 1,700 and 300 Da, broadening the initial mass distribution. Since aggregation and polymerization are known to be competing mechanism and occur simultaneously, it is expected to retrieve lignin fragments of smaller and higher molecular weight that the initial lignin sample.

Secondly, Indulin AT is characterized by large polymers around 21 000 Da. Unlike HE′, EI and HI attest of residual polymers around 6,000 Da. I type of lignin seems to interact less with endogenous lignin than similar lignin such as E and H together after the second treatment. Finally, it appears that E lignin is more sensitive to prolonged treatment once extracted from the plant tissue than H. In addition to be more disposed to cleavage, maximum sizes before and after second treatment are respectively of 60 000 and 170 000 Da attesting of extended aggregation. It can be drawn that, aggregation is likely to take place, involving an increase in the heterogeneity of lignin. However, as highlighted by the NMR spectra, the molecular rearrangement occurs through the formation of common linkages such as β-O-4′ for the ether bond and C-C in resinol substructures.

### Valorization Perspectives

Several assumptions can be drawn regarding some potential valorization ways for metabolites resulting from this treatment, leading to some perspectives for further researches.

Firstly, the residual solid fraction of the mixed feedstock has been enriched in cellulose after the alkali pretreatment, reaching 57.1% while starting raw material contain between 27 and 28% of cellulose. In addition, mixing biomass allowed to recover more cellulose than theoretically (94% > 80%), especially by recovering more cellulose from E highlighting a possible cellulose valorization from *Euphorbia lathyris* in mixed feedstock approach rather than by the single feedstock approach. Moreover, as explained above in the manuscript, the selected pretreatment allows the partial hemicellulose and lignin removal. The residual pulp has therefore an increased specific area and an improved enzymatic accessibility. Among the different applications considered for the cellulosic fraction, the purpose of energy production is interesting in this biorefining case (Wawro et al., 2019). Indeed, the alkali pretreatment improves the conversion of cellulose to fermentable sugars, used for the production of renewable ethanol. (Das et al., 2017) compared alkali and acid pretreatment on different biomass such as hemp fibers. They showed that NaOH pretreatment on hemp allows an increase of the yield of glucose from 25 to 96% compared to native biomass. Moreover, the theatrical ethanol yield obtained after sugars fermentation (310 L/dry ton of biomass) are similar using alkali or dilute acid pretreatment.

Secondly, regarding lignin, our results highlight that biomass ratio and pretreatment conditions can be modulate according to the desired lignin value.

On one hand, by increasing euphorbia content, protein contamination will increase as well and a prolonged treatment will be needed to decrease impurities while promoting interunit linkages condensation (C-C) as a side effect. However, it is worth noting that condensation reaction occur preferentially involving the C5 of the phenylpropane unit. The use of angiosperm dicotyledon feedstock like hemp and euphorbia allow to produce a lignin enriched in syringyl units (S units). S units have a -OCH_3_ moiety at the C3 and C5 of the phenylpropane structure leading to the unavailability of C5 to be involved in the formation of new C-C bond and therefore mitigate the condensation (Kim and Kim, 2018).

On another hand, by increasing hemp content in the ratio, carbohydrates impurities will increase as well, regardless of the pretreatment time. While looking at FTIR of H, HE lignin after the first thermic treatment tend to indicate a relatively similar content in ether bonds. Observation can be compared with 2D HSQC NMR spectra which reveal roughly similar intensities for signals arising from β-O-4′ substructures. Ether bonds are the most easily cleavable bonds amongst lignin linkages (Chakar and Ragauskas, 2004) and have therefore a predominant role in valorization pathways that involve depolymerization reactions. Therefore, increasing hemp content while keeping similar pretreatment time and temperature will lead to mid-value lignin containing polysaccharides contamination, low protein impurities and wide amount of cleavable linkages for depolymerization.

Finally, alkaline lignin from non woody biomass with relatively high rate of aryl ether linkages are also considered as potential raw material for pyrolysis processes since their activation energy is the lowest amongst the different botanical origins and are likely to be decomposed, forming products including phenol and phenolics (Jiang et al., 2010).

## Conclusion

Our study has proven the feasibility of biorefining of hemp and euphorbia during a mixed-feedstocks alkaline treatment. The combination of the biomasses enhanced the lignin recovery while decreasing the contamination arising from carbohydrates and proteins. Besides, it is worth noting that cellulosic residue is similar to the ones obtained after single feedstock pretreatments of hemp and euphorbia.

Lignin FTIR, TGA, GPC and NMR analyzes did not reveal any significant interaction between lignins from two different biomasses during alkaline treatment. The identification of the biomasses used in the multi-feedstock treatment as well as their proportion in the mix could enable to forecast the nature and the property of the resulting ligneous fraction. However, the study of a similar treatment performed on extracted lignin mimicking a prolonged treatment revealed an increase in its molecular mass whether in the case of single or mixed feedstocks. Alteration of the thermal properties occurs as well during the second processing of the lignins. Nevertheless, the comparison of HSQC-NMR analyses of the lignins from the two thermic treatment showed that no linkage of a different nature were formed, suggesting a molecular rearrangement that occurs through the formation of linkages already encountered in the single processed lignin such as β-O-4′ for the ether bond and C-C in resinol substructures.

A comparison of the generated products with the current raw materials used for biobased applications have allowed to draw some assumption of valorization perspectives. Cellulosic pulp is assumed to be quite suitable for fermentation application as was produced by an alkaline treatment which is known to enhance enzymatic digestibility of carbohydrates. Lignin was considered as a potential candidate for depolymerization processes and pyrolysis as it contains significant amount of aryl ether bonds and it is enriched in S units thank to the botanical origin of the feedstocks. Nevertheless, in a perspective of gathering multiple sources of biomass in a mixed feedstock biorefinery approach, a particular attention needs to be drawn on the origin and the proportions of the biomasses since it can result in monolignols composition and a wide variation of lignin purity. The later parameters can be partially controlled by adapting process condition, involving as well a modification in lignin structural characteristics.

## Data Availability Statement

The datasets generated for this study are available on request to the corresponding author.

## Author Contributions

TB, QS, and TL designed the analytical procedures of lignin characterization and interpreted the results. QS designed the experimental plan and performed pretreatments concerning mass balance investigation. TB and QS wrote the manuscript. AR obtained the research funds. All authors approved the final version.

## Conflict of Interest

The authors declare that the research was conducted in the absence of any commercial or financial relationships that could be construed as a potential conflict of interest.
