# Supplementary material for: Single and Mixed Feedstocks Biorefining: Comparison of Primary Metabolites Recovery and Lignin Recombination During an Alkaline Process
Source: Front Chem. 2020 Jun 5;8:479. doi: 10.3389/fchem.2020.00479 (PMC7292014; doi:10.3389/fchem.2020.00479)
Supplement: Supplementary file 1 [file Image_1.pdf]

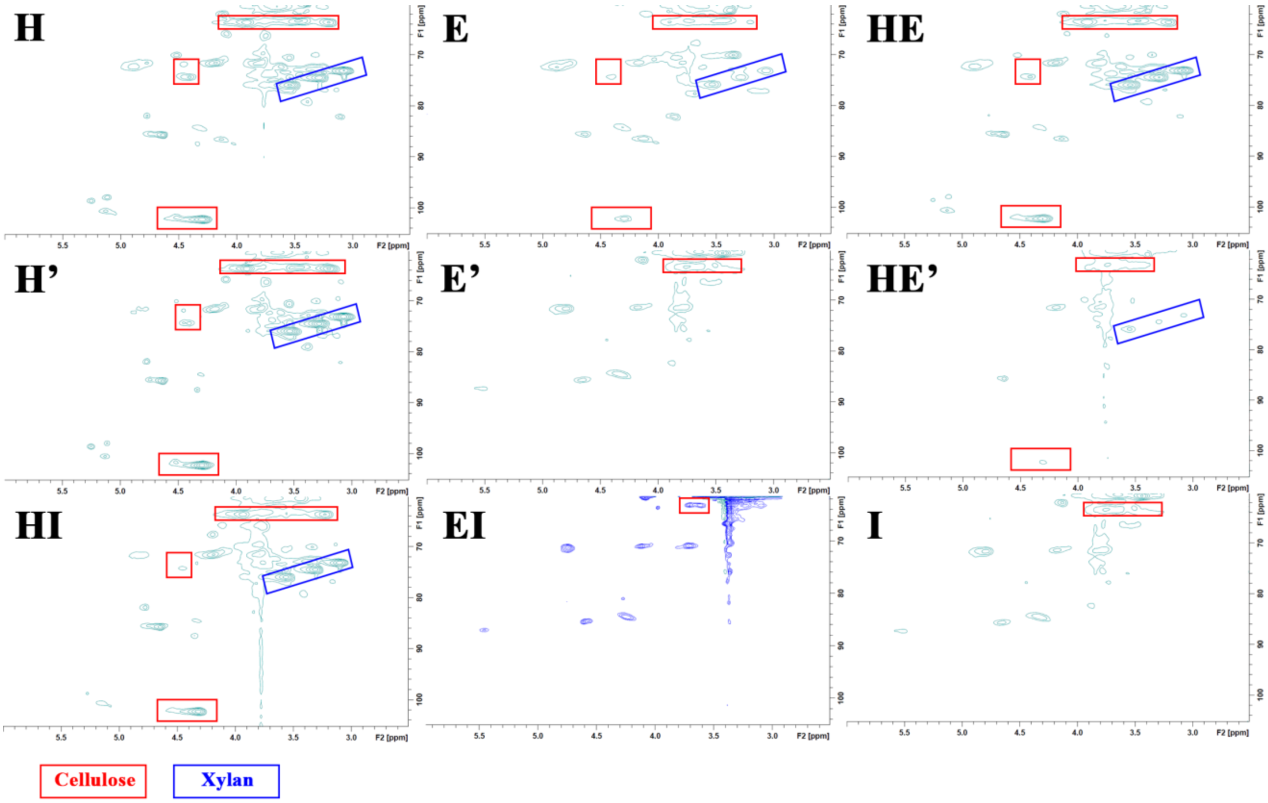

Figure S1: 2D HSQC NMR spectra of carbohydrate moieties according to the chemical shift area proposed by Jiang et al., 2018
