# Supplementary material for: Single and Mixed Feedstocks Biorefining: Comparison of Primary Metabolites Recovery and Lignin Recombination During an Alkaline Process
Source: Front Chem. 2020 Jun 5;8:479. doi: 10.3389/fchem.2020.00479 (PMC7292014; doi:10.3389/fchem.2020.00479)
Supplement: Supplementary file 2 [file Image_2.pdf]

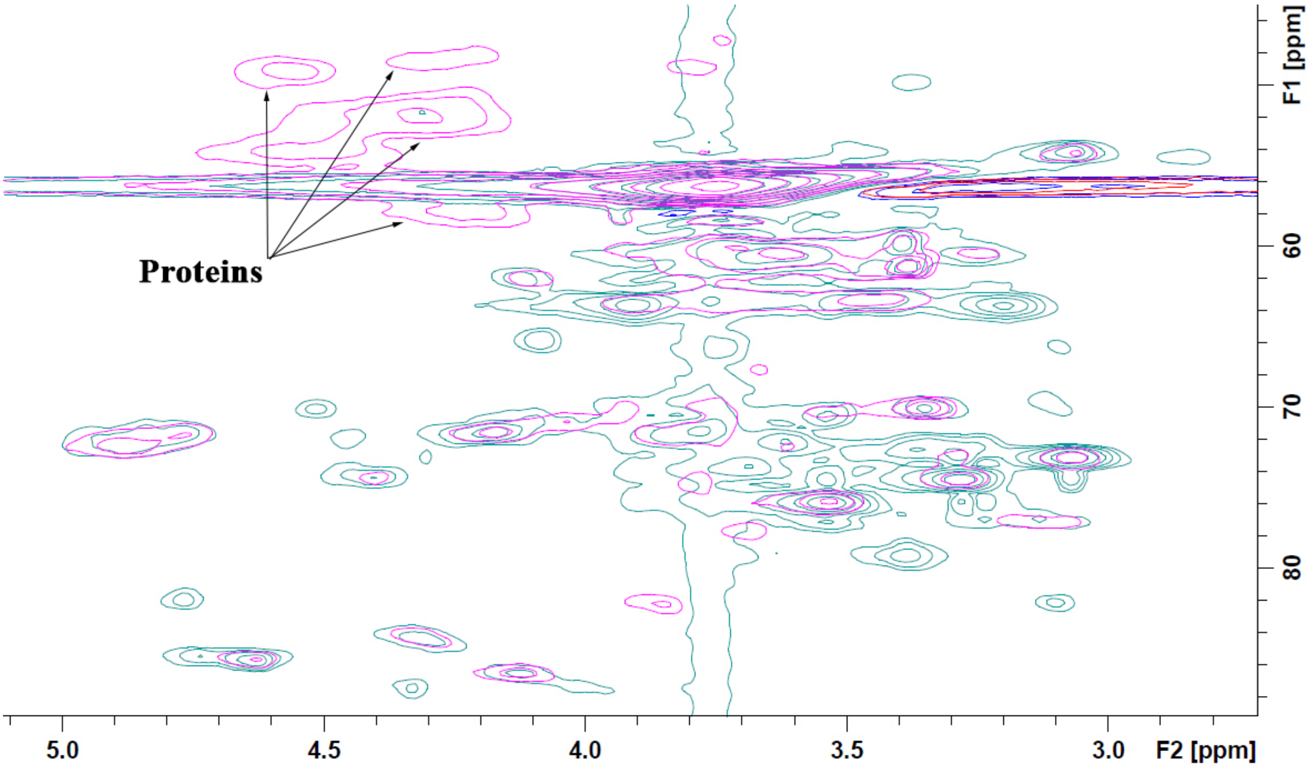

Figure S2: Overlaying of lignin 2D HSQC NMR spectra of E (pink) and E’ (green) that highlight the presence of some proteins in the lignin recovered after the first thermic treatment only according to the chemical shift area proposed by Liitia et al., 2003
